# Supplementary material for: Genomic Variation Influences Methanothermococcus Fitness in Marine Hydrothermal Systems
Source: Front Microbiol. 2021 Aug 20;12:714920. doi: 10.3389/fmicb.2021.714920 (PMC8417812; doi:10.3389/fmicb.2021.714920)
Supplement: Supplementary Figure 1 — Box-and-whisker and swarm plots indicating the metatranscriptomic coverage of specific genes in Methanothermococcus single cell-amplified genomes in sample FS848, Ginger Castle, at Von Damm hydrothermal field. Coverage values are not normalized between SAGs, and thus relative transcript abundance of specific gene types should only be compared within individual SAGs. The boxes show the quartiles of the dataset, and the whiskers extend to show the rest of the distribution. Outliers are indicated by points outside of the whiskers. Individual dots indicate individual gene coverage values for genes within that category. Y axes are split to depict the full range of coverage values. A list of the KEGG numbers that were included within each category is listed in Supplementary Data File 1. [file Image_1.pdf]

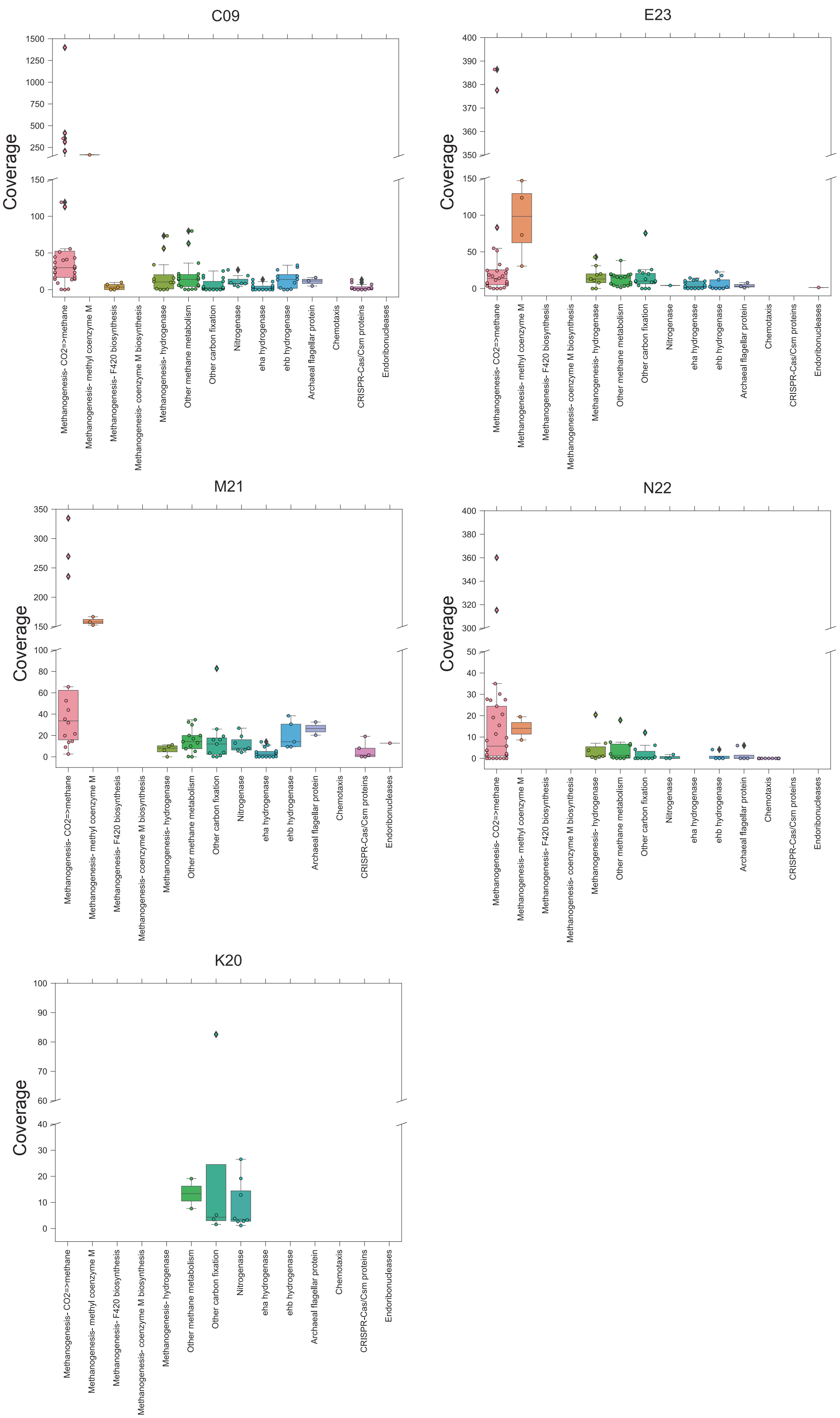


**Supplementary Figure 1.** Box-and-whisker and swarm plots indicating the metatranscriptomic coverage of specific genes in *Methanothermococcus* single cell-amplified genomes in sample FS848, Ginger

Castle, at Von Damm hydrothermal field. Coverage values are not normalized between SAGs, and thus relative transcript abundance of specific gene types should only be compared within individual SAGs. The boxes show the quartiles of the dataset, and the whiskers extend to show the rest of the distribution. Outliers are indicated by points outside of the whiskers. Individual dots indicate individual gene coverage values for genes within that category. Y axes are split to depict the full range of coverage values. A list of the KEGG numbers that were included within each category is listed in Supplementary Data File 1.
