# Supplementary material for: Genomic Variation Influences Methanothermococcus Fitness in Marine Hydrothermal Systems
Source: Front Microbiol. 2021 Aug 20;12:714920. doi: 10.3389/fmicb.2021.714920 (PMC8417812; doi:10.3389/fmicb.2021.714920)
Supplement: Supplementary Figure 2 — Summary of CRISPR loci in the 5 Methanothermococcus SAGs from Ginger Castle vent from Von Damm vent field on the Mid-Cayman Rise, sampled in 2012, plus Methanothermococcus okinawensis IH1 for reference. Each of the CRISPR loci is represented by a line with a series of boxes, in which each box represents a spacer sequence. Matching spacers are shown in the same color; unique spacers are shown in black. [file Image_2.pdf]

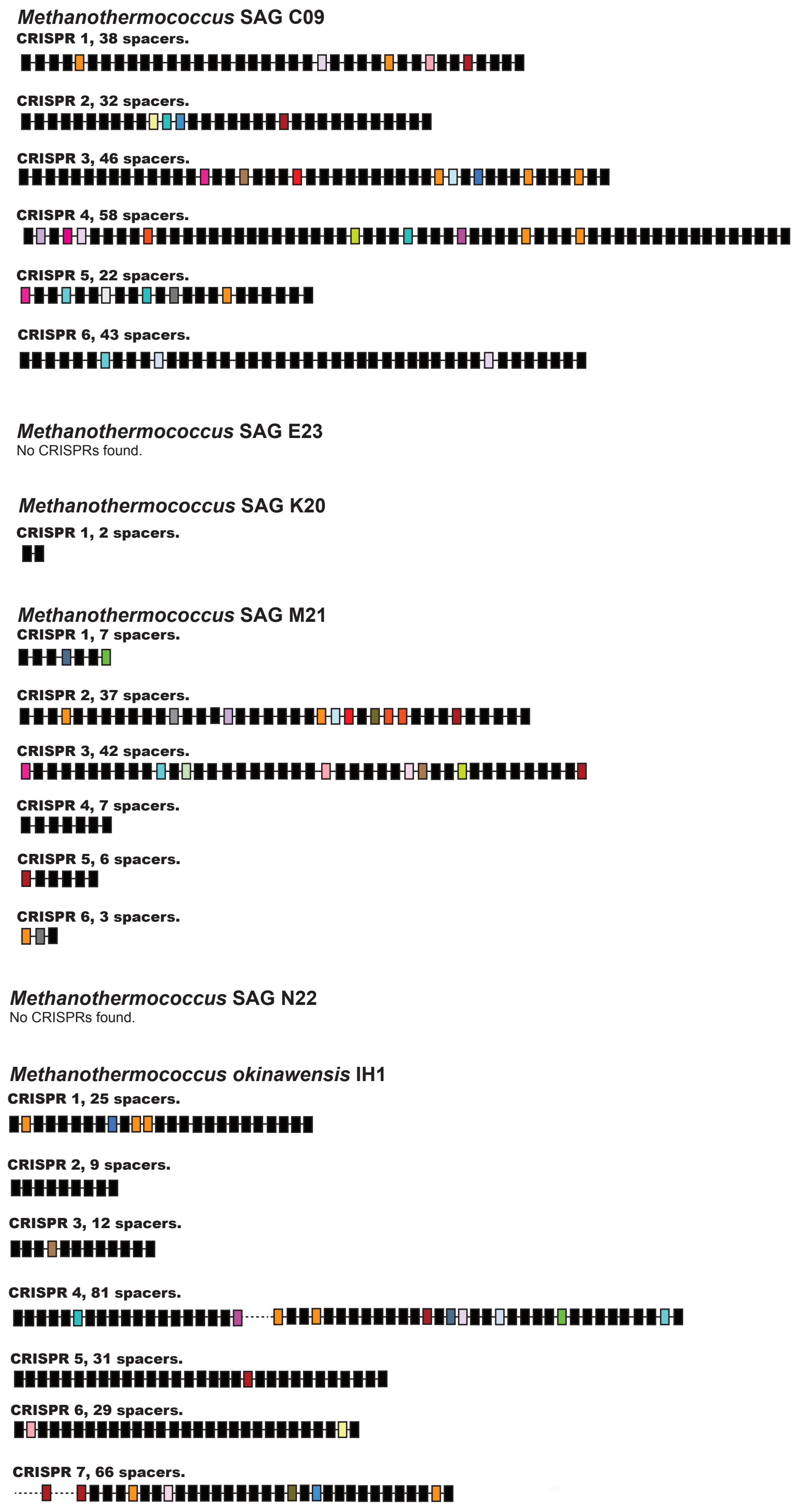
Methanothermococcus SAG C09
CRISPR 1, 38 spacers.
CRISPR 2, 32 spacers.
CRISPR 3, 46 spacers.
CRISPR 4, 58 spacers.
CRISPR 5, 22 spacers.
CRISPR 6, 43 spacers.
Methanothermococcus SAG E23
No CRISPRs found.
Methanothermococcus SAG K20
CRISPR 1, 2 spacers.
Methanothermococcus SAG M21
CRISPR 1, 7 spacers.
CRISPR 2, 37 spacers.
CRISPR 3, 42 spacers.
CRISPR 4, 7 spacers.
CRISPR 5, 6 spacers.
CRISPR 6, 3 spacers.
Methanothermococcus SAG N22
No CRISPRs found.
Methanothermococcus okinawensis IH1
CRISPR 1, 25 spacers.
CRISPR 2, 9 spacers.
CRISPR 3, 12 spacers.
CRISPR 4, 81 spacers.
CRISPR 5, 31 spacers.
CRISPR 6, 29 spacers.
CRISPR 7, 66 spacers.

Supplementary Figure 2. Summary of CRISPR loci in the 5 *Methanothermococcus* SAGs from Ginger Castle vent from Von Damm vent field on the Mid-Cayman Rise, sampled in 2012, plus *Methanothermococcus okinawensis* IH1 for reference. Each of the CRISPR loci is represented by a line with a series of boxes, in which each box represents a spacer sequence. Matching spacers are shown in the same color; unique spacers are shown in black.
