# Supplementary material for: Genomic Variation Influences Methanothermococcus Fitness in Marine Hydrothermal Systems
Source: Front Microbiol. 2021 Aug 20;12:714920. doi: 10.3389/fmicb.2021.714920 (PMC8417812; doi:10.3389/fmicb.2021.714920)
Supplement: Supplementary Figure 4 — Scatterplots of average mapping coverage vs. average number of SNVs per kilobase pair of DNA for metagenomic mappings, colored by SAG. Each point represents data from a different metagenome mapped to each SAG. [file Image_4.pdf]

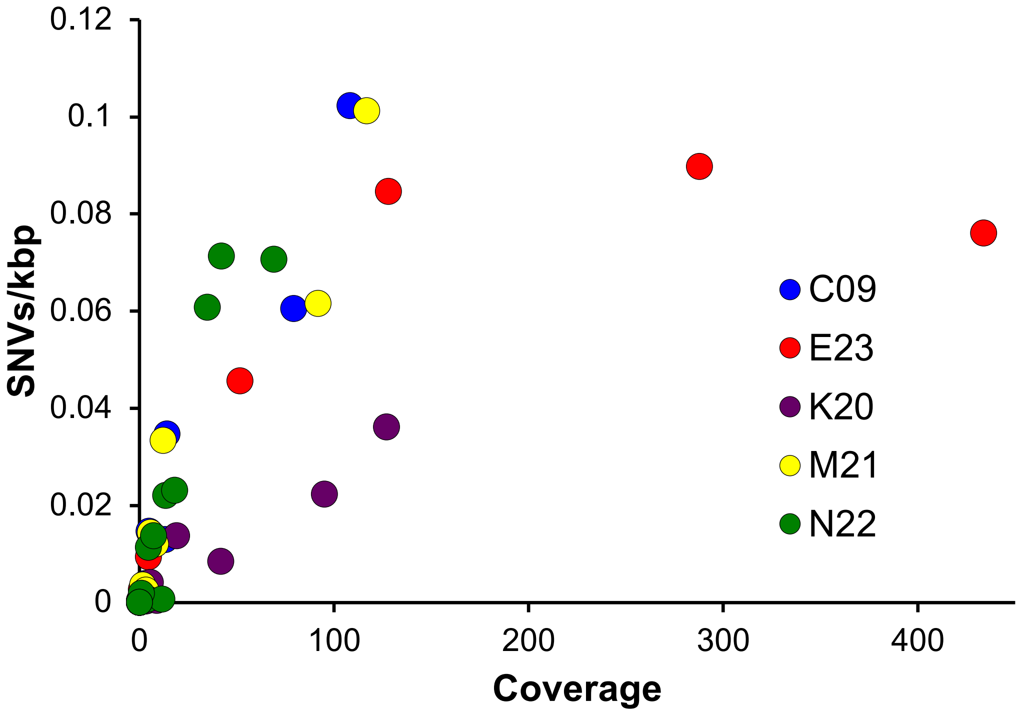

Supplementary Figure 4. Scatterplots of average mapping coverage vs average number of SNVs per kilobase pair of DNA for metagenomic mappings, colored by SAG. Each point represents data from a different metagenome mapped to each SAG.
