# Supplementary material for: Genomic Variation Influences Methanothermococcus Fitness in Marine Hydrothermal Systems
Source: Front Microbiol. 2021 Aug 20;12:714920. doi: 10.3389/fmicb.2021.714920 (PMC8417812; doi:10.3389/fmicb.2021.714920)
Supplement: Supplementary Table 1 — Data regarding quality filtering and assembly of metagenomic and metatranscriptomic reads. No MT, no metatranscriptome. NA, not available due to loss of USBL navigation by the ROV. [file Table_1.docx]

| **Vent Field** | **Vent Name** | **Year** | **Sample Number** | **Latitude** | **Long-itude** | **Temp-erature (˚C)** | **Number of raw meta-genomic reads (pairs)** | **Number of quality filtered meta-genomic reads** | **ENA Sample Accession** | **ENA Secondary Accession** | **Number of raw metatranscriptomic reads (pairs)** | **Number of quality filtered metatranscriptomic reads, rRNA removed** |
| --- | --- | --- | --- | --- | --- | --- | --- | --- | --- | --- | --- | --- |
| Von Damm | Old Man Tree | 2012 | FS841 | 18.375 | -81.8 | 114 | 61008797 | 53,972,022 | ERS1370013 | SAMEA4470834 | 14536191 | 3,427,865 |
| Von Damm | Ravelin #2 | 2012 | FS842 | 18.3752 | -81.8 | 86 | 69963233 | 64,064,672 | ERS1370014 | SAMEA4470835 | no MT | no MT |
| Von Damm | Shrimp Hole | 2012 | FS844 | 18.3747 | -81.8 | 50 | 111467935 | 104,733,336 | ERS1370015 | SAMEA4470836 | 44242399 | 29,768,330 |
| Von Damm | Ginger Castle | 2012 | FS848 | 18.3769 | -81.8 | 47 | 81658568 | 77,403,857 | ERS1370016 | SAMEA4470837 | 25427198 | 6,231,008 |
| Von Damm | Main Orifice | 2012 | FS849 | 18.3767 | -81.8 | 109 | 30239186 | 26,539,513 | ERS1370017 | SAMEA4470838 | no MT | no MT |
| Piccard | Hot Chimlet, BVM | 2012 | FS851 | 18.5472 | -81.72 | 106 | 88561544 | 78,203,317 | ERS1370018 | SAMEA4470839 | 25550688 | 5,385,293 |
| Piccard | Shrimp Canyon, BVM | 2012 | FS852 | 18.5471 | -81.72 | 44 | 42204178 | 38,058,339 | ERS1370019 | SAMEA4470840 | 26316504 | 10,571,338 |
| Piccard | X-19 at BV #4, BVM | 2012 | FS854 | 18.5466 | -81.72 | 18 | 148334278 | 141,073,647 | ERS1370020 | SAMEA4470841 | 12643619 | 1,126,747 |
| Piccard | Shrimp Gulley #2, BSM | 2012 | FS856 | 18.5466 | -81.72 | 108 | 109104481 | 104,273,127 | ERS1370021 | SAMEA4470842 | 11471361 | 3,822,920 |
| Von Damm | near Main Orifice | 2013 | FS866 | 18.3767 | -81.8 | 130 | 27575200 | 25,829,542 | ERS1370022 | SAMEA4470843 | no MT | no MT |
| Von Damm | Shrimp Hole | 2013 | FS872 | 18.3747 | -81.8 | 30 | 45935925 | 40,627,197 | ERS1370023 | SAMEA4470844 | 22530122 | 19,601,515 |
| Von Damm | Twin Peaks | 2013 | FS874 | NA | NA | 140 | 72719556 | 67,240,297 | ERS1370024 | SAMEA4470845 | no MT | no MT |
| Von Damm | Shrimp Buttery | 2013 | FS877 | NA | NA | 131 | 167341615 | 158,036,033 | ERS1370025 | SAMEA4470846 | no MT | no MT |
| Von Damm | Hot Cracks #2 | 2013 | FS879 | 18.375 | -81.8 | 29 | 27319409 | 25,899,380 | ERS1370026 | SAMEA4470847 | 21345009 | 8,229,809 |
| Von Damm | Old Man Tree | 2013 | FS881 | 18.375 | -81.8 | 114 | 191547095 | 178,390,689 | ERS1370027 | SAMEA4470848 | 14231356 | 8,856,348 |

**Supplementary Table 1.** Data regarding quality filtering and assembly of metagenomic and metatranscriptomic reads. No MT, no metatranscriptome. NA, not available due to loss of USBL navigation by the ROV.
