# Supplementary material for: Genomic Variation Influences Methanothermococcus Fitness in Marine Hydrothermal Systems
Source: Front Microbiol. 2021 Aug 20;12:714920. doi: 10.3389/fmicb.2021.714920 (PMC8417812; doi:10.3389/fmicb.2021.714920)
Supplement: Supplementary Table 2 — Percent reads mapping for all SAG-to-SAG mappings. SAG contigs used as reference are listed on the left, SAG reads mapped to the reference are listed along the top of the table. Numbers are listed as percent of merged reads mapped using bowtie2. [file Table_2.docx]

|  | **SAG reads** | | | | | |
| --- | --- | --- | --- | --- | --- | --- |
| **SAG reference** |  | **C09** | **E23** | **K20** | **M21** | **N22** |
|  | **C09** | 95.82 | 0 | 33.83 | 64.14 | 0.1 |
|  | **E23** | 0 | 93.43 | 0 | 0 | 1.03 |
|  | **K20** | 4.6 | 0 | 97.7 | 2.11 | 0 |
|  | **M21** | 42.44 | 0 | 18.3 | 96.53 | 0.05 |
|  | **N22** | 1.09 | 2.15 | 1.23 | 0.08 | 65 |

**Supplementary Table 2**. Percent reads mapping for all SAG-to-SAG mappings. SAG contigs used as reference are listed on the left, SAG reads mapped to the reference are listed along the top of the table. Numbers are listed as percent of merged reads mapped using bowtie2.
