# Supplementary material for: Genomic Variation Influences Methanothermococcus Fitness in Marine Hydrothermal Systems
Source: Front Microbiol. 2021 Aug 20;12:714920. doi: 10.3389/fmicb.2021.714920 (PMC8417812; doi:10.3389/fmicb.2021.714920)
Supplement: Supplementary Table 3 — Selected gene counts for genes present and/or absent in each SAG from the Mid-Cayman Rise. [file Table_3.docx]

| **Gene name** | **Category** | **C09** | **E23** | **K20** | **M21** | **N22** | |
| --- | --- | --- | --- | --- | --- | --- | --- |
| iron transport protein (FeoB_CN) | **Fe** | 2 | 3 | 0 | 0 | 2 | |
| iron transport protein (FeoA) | **Fe** | 3 | 4 | 0 | 1 | 2 | |
| ech hydrogenase (echABCE) | **H_2_** | 0 | 0 | 0 | 0 | 0 | |
| energy-converting hydrogenase A (ehaBCEGNOP) | **H_2_** | 28 | 24 | 0 | 20 | 10 | |
| energy-converting hydrogenase B (ehbABFIJKLNO) | **H_2_** | 28 | 24 | 0 | 20 | 10 | |
| coenzyme F420 hydrogenase (frhAGDG) | **H_2_** | 2 | 2 | 0 | 1 | 2 | |
| hydrogenase (hyaABC, hybO, hybC) | **H_2_** | 0 | 0 | 0 | 0 | 0 |  |
| [NiFe] hydrogenase (hydA2A3B2B3) | **H_2_** | 0 | 0 | 0 | 0 | 0 |  |
| methane/ammonia monooxygenase (pmoA-amoA) | **CH_4_** | 0 | 0 | 0 | 0 | 0 |  |
| methyl-coenzyme M reductase (mcrABCDG) | **CH_4_** | 3 | 3 | 0 | 3 | 4 |  |
| cytochrome c oxidase (coxABCD,AC,ctaF) | **O_2_** | 0 | 0 | 0 | 0 | 0 |  |
| cytochrome c oxidase cbb3-type (ccoNOPQ_NO) | **O_2_** | 0 | 0 | 0 | 0 | 0 |  |
| carbon-monoxide dehydrogenase (cooCFS, acsA) | **O_2_** | 2 | 2 | 0 | 0 | 0 |  |
| fumarate reductase (frdAB) subunitAB | **C** | 2 | 2 | 1 | 2 | 2 |  |
| Nitrogenase | **N** | 1 | 0 | 1 | 1 | 0 |  |
| Phosphoadenosine phosphosulfate reductase/PAPS reductase | **S** | 1 | 1 | 0 | 1 | 0 |  |
| Serine O-acetyltransferase (cysE) | **S** | 1 | 1 | 1 | 0 | 1 |  |
| Chemotaxis proteins (M. okinawensis 1415, 1417, 1418; chemotaxis proteins CheW, CheY and CheA; methyl-accepting chemotaxis signaling domain-containing protein, methyl-accepting chemotaxis sensory transducer) | **Chemo-taxis** | 0 | 0 | 0 | 0 | 7 |  |
| Cas proteins (cas1-6) | **CRISPR** | 6 | 0 | 0 | 0 | 0 |  |
| Csm proteins (csm1-5) | **CRISPR** | 5 | 0 | 0 | 5 | 0 |  |

**Supplementary Table 3.** Selected gene counts for genes present and/or absent in each SAG from the Mid-Cayman Rise.
