# Supplementary material for: Genomic Variation Influences Methanothermococcus Fitness in Marine Hydrothermal Systems
Source: Front Microbiol. 2021 Aug 20;12:714920. doi: 10.3389/fmicb.2021.714920 (PMC8417812; doi:10.3389/fmicb.2021.714920)
Supplement: Supplementary Figure 3 — Plot of SNVs per kilobase pair vs. n2n1 (majority allele frequency) for metagenome mappings to single cell amplified genomes from Piccard (yellow) and Von Damm (blue) vent fields. All y-axes reflect n2n1, or the ratio of the second most common allele to the most common allele. Bubble diameter corresponds to the average coverage of each SAG-metagenome mapping normalized by number of metagenomic reads. Bubbles are colored according to which vent field they came from; sample numbers are indicated. [file Image_3.pdf]

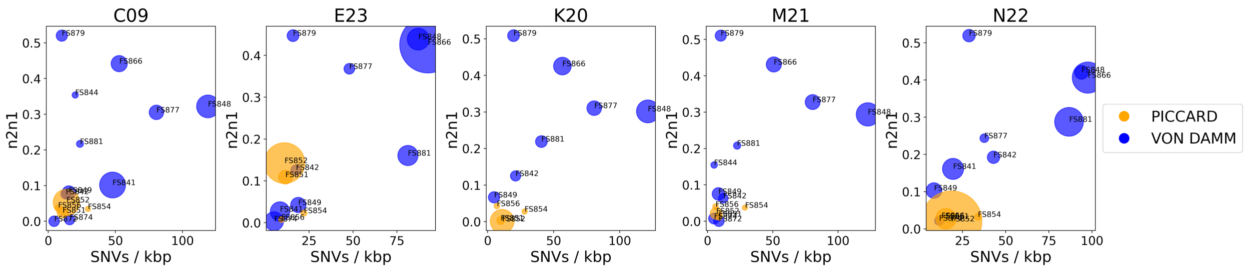

Supplementary Figure 3. Plot of SNVs per kilobase pair versus n2n1 (majority allele frequency) for metagenome mappings to single cell amplified genomes from Piccard (yellow) and Von Damm (blue) vent fields. All y-axes reflect n2n1, or the ratio of the second most common allele to the most common allele. Bubble diameter corresponds to the average coverage of each SAG-metagenome mapping normalized by number of metagenomic reads. Bubbles are colored according to which vent field they came from; sample numbers are indicated.
